# Stromal microenvironment processes unveiled by biological component analysis of gene expression in xenograft tumor models

**DOI:** 10.1186/1471-2105-11-S9-S11

**Published:** 2010-10-28

**Authors:** Xinan Yang, Younghee Lee, Yong Huang, James L Chen, Rosie H Xing, Yves A Lussier

**Affiliations:** 1 Section of Genetic Medicine, Dept. of Medicine, University of Chicago, IL, USA; 2 Sect. of Hematology/Oncology, Dept. of Medicine, University of Chicago, IL, USA; 3 Dept. of Pathology and of Radiation and Cellular Oncology; University of Chicago, IL, USA; 4 UC Comprehensive Cancer Centre; Ludwig Centre for Metastasis Research; University of Chicago, IL, USA; 5 Institute of Genomics and Systems Biology; Institute for Translational Medicine; Computational Institute, University of Chicago, IL, USA

## Abstract

**Background:**

Mouse xenograft models, in which human cancer cells are implanted in immune-suppressed mice, have been popular for studying the mechanisms of novel therapeutic targets, tumor progression and metastasis. We hypothesized that we could exploit the interspecies genetic differences in these experiments. Our purpose is to elucidate stromal microenvironment signals from probes on human arrays unintentionally cross-hybridizing with mouse homologous genes in xenograft tumor models.

**Results:**

By identifying cross-species hybridizing probes from sequence alignment and cross-species hybridization experiment for the human whole-genome arrays, deregulated stromal genes can be identified and then their biological significance were predicted from enrichment studies. Comparing these results with those found by the laser capture microdissection of stromal cells from tumor specimens resulted in the discovery of significantly enriched stromal biological processes.

**Conclusions:**

Using this method, in addition to their primary endpoints, researchers can leverage xenograft experiments to better characterize the tumor microenvironment without additional costs. The Xhyb probes and R script are available at http://www.lussierlab.org/publications/Stroma

## Background

Characterizing the tumor microenvironment is essential as it relates to clinical prognoses, metastatic potential, and treatment-related outcomes [1]. Innovations in cell labeling techniques and small-animal *in vivo* imaging have enabled investigators to phenotype the microenvironment and cancer cell interactions. However, genome-wide expression analyses of the tumor and its microenvironment have not kept pace. Tearing apart cancer cells from stromal tissues in whole tissue expression requires time consuming and costly laser microdissection of the tumor before RNA extraction [2,3]. Computational methods previously have been proposed as a means of subtraction out the stromal signal [4]. Yet this method simply eliminates genes which have conflated expression levels, however it does little to elucidate the tumor microenvironment.

In this paper, we describe a computational method of exploiting interspecies differences in the mouse tumor xenograft model in which human cancer cells are grown in immune-suppressed mice. This model is popular for studying the mechanisms of preclinical drug trials, tumor progression and the development of metastasis. Mouse xenografts consist of both human tumor cells and mouse stromal tissues [5], thus differential gene expression derived from human expression arrays are generally implicitly attributed to the cancer cells [6,7]. However, cross-hybridization of human chip probes with homologous mouse genes may result in a mixed gene expression signal where deregulated mice stromal genes in xenograft tumor models are being jointly measured along with the human cancer genes. Up to date, functional categories (Gene Ontology terms) enriched in the gene expression of whole tumor xenografts have neglected the impact of cross-species hybridization.

Tumor/stromal interactions have been examined by modeling the diffusion of the nutrients in the stroma, invasion of single cells into the stroma, and characterizing the function of the stromal elements [8-10]. To our knowledge, no studies have specifically focused on identifying mouse stromal signals in human gene arrays. However, unsurprisingly, a few groups have identified cross-species signals in multiple species chips [11,12]. Others and us have designed pan-viral arrays comprising of species-probes for over 1000 species on one chip for diagnostic purposes [12,13]. In collaboration with others, we have demonstrated that statistically-based gene enrichment approaches to these pan-viral arrays were effective in increasing the species-specific signal and consequently the diagnostic accuracy of these pan-microbial arrays [14]. The xenograft tumor expressed on human arrays presents a multiple species problem. Human probes were not designed to be species specific and thus, the cross-hybridization is equally present in Human expression arrays (if not more important) than in pan-microbial arrays.

We hypothesized that we could identify stromal microenvironment signals (Gene Ontology (**GO**) biological processes) from deregulated genes using the human array probes unintentionally cross-hybridizing with the mouse homolog. This assumption is based on the observation that **1)** the majority of the cross-hybridizing probes designed for human genes also target their mouse homolog, and **2)** the majority of Gene Ontology [15] annotations for human and mouse homolog are identical (Additional file 1: Suppl. Methods). Thus, in this paper, we design an unbiased method to identify and optimize the choice of cross-species hybridizing probes. To test out this method, we compared the Gene Ontology terms enriched in cross-hybridizing probes with those derived from microdissected stromal components.

## Methods

**Figure 1:**
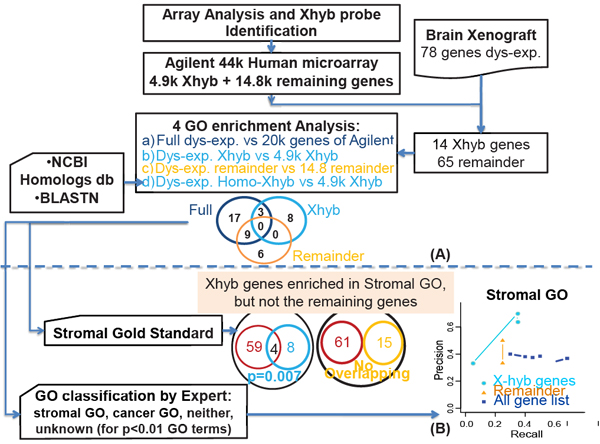
**Description of the Biological Component Analysis Part A**: To derive the possible contribution of the stromal microenvironment from the cross-hybridizing probes, we studied gene lists derived from xenograft models from the literature that used the same microarray platform. Robust GO enrichment analyses[20] were conducted over (i) all genes differentially expressed between the experimental conditions, (ii) the cross-hybridizing subset, or (iii) the remaining genes (**A**, Top Venn Diagram). **Part B:** We evaluated the cross-hybridizing genes enriched GO terms with expert classified GO terms and with the gold standards from literature (Bottom Venn Diagrams). **Legend:** Hs: *Homo sapiens*; Mm: *Mus musculus;* P: Probe; GS: gold standard.

We developed a two-stage method for deriving the underlying biology of the tumor stromal microenvironment that we call **“biological component analysis”.** It is designed to enrich the gene expression signals in the stromal component separately from the cancer cell component of the tumor xenograft (Figure 1).

### Datasets

The GO annotations and gene homolog for the human genome were downloaded from the NCBI. Microarray platform information for the Agilent 44k whole human genome oligo microarray was obtained from the Gene Expression Omnibus [16] (GEO:GPL6480; Additional file 1: Suppl. Methods, Table A).

### Simulating a xenograft model with human and mouse RNA

In order to biologically determine cross-hybridizing probes, the human and mouse universal reference RNAs were obtained from ArrayIt (Sunnyvale, CA).

### Array selection

For this study, we selected the Agilent 44k whole human genome oligo microarray which allows for custom design. The Agilent microarray is designed with one or more 60-mer probes per gene rather than the more complex Affymetrix probe-sets containing multiple shorter (25-mer) probes voting for each gene (Additional file 1: Suppl. Methods, array preprocessing).

### Determining probes of human arrays cross-hybridizing with mouse RNA and the proportion of homologous genes between species

Cross-species hybridizing (**Xhyb**) probes between human and mouse RNA were determined by combining a computational method (BLAST [17]) and a biological method in which the channels of two arrays were independently exposed to either **(i)** mouse RNA, **(ii)** human RNA, or **(iii)** both. For simplicity, these genes are hereafter referred to as **“Xhyb genes”** whose probes were highly expressed when exposed to mouse universal RNAs but lower when exposed to human universal RNAs. We hypothesized that the mouse-specific, and thus stromal-specific, signals are enriched in these cross-species hybridizing probes. From the identified genes with only cross-species hybridizing probes, the proportion of homologous genes between the two species were calculated. This last step was important to justify the GO enrichment studies conducted over cross-hybridizing probes of the human array that were used to produce sets of GO terms describing the biological processes in the stroma (Additional file 1: Suppl. Methods). The normalization model of microarray gene expression on a log scale, noted as *y*, in this xenograft model is:

, (1)

where *μ* represents the overall mean value for a probe, *i,* originally designed for its *Homo sapiens* (*Hs*) target (noted as *μ_i_Hs__*) or *Mus musculus* (*Mm*) homolog if cross-hybridizing (noted as *μ_i_Mm__*). The notation *c* is the main effect of a clinical phenotype, *j*, additive to the probe expression. The notation *ε* is the stochastic error [18] that includes same-species cross-species hybridizing, non-homologous cross-species hybridizing, and other technological errors. Because the four-array experiment was performed under the exact same conditions, we ignored the array effect and array interaction effect in the data extracted by the Agilent Feature Extraction Software for the Agilent 4x44k array (4 arrays per slide). Thus, in this case of cross-species hybridization, we can assume that the distribution of the term (*μ_i_Hs__*+ *c_j_HS__*) is stochastic (centered at zero) and thus, part of the error *ε.* Consequently, the equation for the expression of a cross-hybridizing probe becomes:

. (2)

Where *ε*’ represents stochastic errors. Therefore, the differential expression of a Xhyb probe between two phenotypes, *a* and *b,* is really a stromal gene response rather than a cancer cell response on a log scale:

. (3)

**Figure 2:**
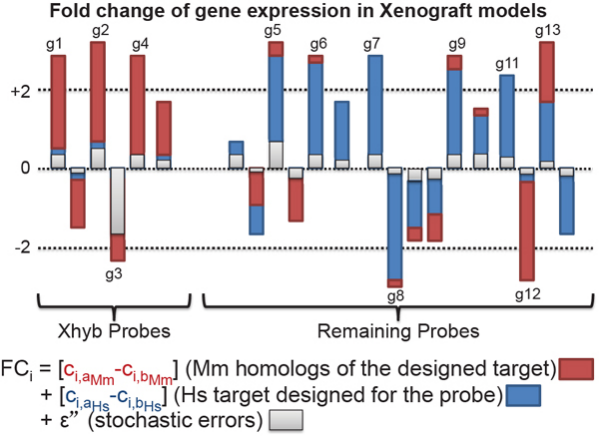
**A simulated visual model of differential genes expression in a xenograft experiment**. The numbers in the plot are the simulated gene symbols of differentially expressed probes. **Legend**: a, b: two sample groups; c: effect of interested; i: probe id; FC: fold change (**Equation. 1**).

We make standard stochastic assumptions about the errors *ε, ε’* and *ε”*. As shown in Figure 2, when Xhyb human probes are exposed to both human and mouse RNA, their major expression levels reflect the expression of their homologous mouse genes. Thus, the GO terms enriched in these cross-species hybridizing human gene targets represent the stromal response of their homologous mouse genes. In contrast, in the remaining deregulated genes (**remaining genes**) that are putatively enriched in cancer cell signal, the majority of gene expressions were contributed by designed targets which reflect the tumor biological processes of human tumor tissues.

A stromal GO term is contributed by genes where cross-species hybridization is more likely to occur as presented in Table 1. Thus by selecting genes that are differentially expressed between phenotypes and also Xhyb, we identify a stromal-specific GO term that can distinguish among phenotypes. This is because each of the hybridizing probes of the human array expresses only when it is exposed to mouse RNA in our biological experiment. Thus the differentially expressed probe between phenotypes reflects the dysregulation of its homologous mouse target rather than the stochastic effect or cancer effect.

**Table 1:** Deregulated human genes and their contributed Gene Ontology in a xenograft model.

Gene Ontology Enrichment from the Differentially Expressed Genes	Expression of Significantly Differentially Expressed Probes in Human Array	
	
	Xhyb probes	Remainders	
Biological process chiefly attributable to mouse stromal cells	*c_i,j_Mm__* » *c_i,j_Hs__*	*c_i,j_Hs__* is stochastically distributed
Biological process chiefly attributable to human cancer cells	*c_i,j_Mm__* is stochastically distributed	*c_i,j_Mm__* « *c_i,j_Hs__*
Biological processes enriched in the experiment	Both *c_i,j_Mm__* and *c_i,j_Hs__* are stochastically distributed	

This table describes the relationship between deregulated genes and their contributed Gene Ontology in a xenograft model, using human microarray. **Legend**: *c_i,j_Hs__* is the main effect of a clinical phenotype *j* on a *Homo sapiens (Hs)* gene targeted by a probeset *i*; where *c_i,j_Mm__* is the main effect of a clinical phenotype *j* on a *Mus musculus* (*Mm*) gene cross-hybridized by a probeset *i*.

### Stromal effect among literature-reported stroma gene lists (Figure 1A, Additional file 2: Suppl. Table 1)

Xenograft models of human cancer and mouse stroma are generally analyzed using human arrays for deregulated genes, and the expression profiles are generally attributed to the human gene's biological processes or functions. To assess the stromal effect among the reported tumor contributing genes, we searched the PubMed resource by keywords “xenograft, Agilent, cancer, gene expression”. Two of the resulting publications fit the criteria using the same platform (GEO:GPL6480) as shown in Table 2. Among these two publications, a glioblastoma [19] study was selected for further GO enrichment analysis as it contained enough “literature-reported genes” (Additional file 2: Suppl. Table 1). The literature-reported genes, whose probes cross-hybridize with mouse RNAs (Xhyb), were thereafter searched using the available GenBank IDs or official gene symbols.

**Table 2:** Previous xenograft cancer studies using the Agilent GPL6480 whole human microarray.

Cancer	Sample	Reported Genes
Glioblastomas (GBM)[20]	10 GBMs xenografts	78 genes differentially expressed between the first and later GBM xenograft generations.
Colon cancer[6]	3-4 xenografts per group	28 genes deregulated in mutant tumor xenografts versus the control tumor xenografts.

### Tests to determine the statistical significance of enriched cross-species GO terms

To identify the overrepresented biological processes, the Entrez Gene ID for annotated probes were inputted into the Bioconductor package *GOstats *[20], followed by the enrichment of GO biological process (BP) terms conducted in four ways. In **Enrichment a** (conventional enrichment of genes derived from Xenograft models), the list of literature-reported genes (defined in previous paragraph, Additional file 2: Suppl. Table 1) were compared to the entire gene set of the 44k Agilent microarray. In **Enrichment b** (enrichment of putative stromal signal), the Xhyb subset of the literature-reported genes were compared to all Xhyb genes in the microarray that we had identified. In **Enrichment c** (enrichment of cancer cells signal), the remaining (non-Xhyb) subset of reported genes were compared to the remaining (non-Xhyb) genes in the microarray. In **Enrichment d** (enrichment of more specific stromal signal), the subset of reported genes whose probes cross-hybridized with their mouse homolog were compared to all the genes with cross-hybridized probes on the array. Note that since we could not know which cross-hybridizing probes targeted which homolog on the entire array without experimental evidence, test *d)* used all genes with cross-hybridizing probes as the background resulting in an estimated statistic. Alternatively, approach *b)* uses an evaluation by proxy based on the observation that the majority of mouse GO Annotations were identical to human GO Annotations, and the majority of the cross-hybridizing genes target their corresponding mouse homolog (Additional file 1: Suppl. Methods). For each described Tests (above Tests “a”, “b”, “c’, “d”), a “conditional” enrichment analysis [20] was conducted. This “conditional” hypergeometric test allows for (i) controlling false positives results resulting from genes inherited in the hierarchical structure of GO instead (ii) increasing the robustness of results from small gene lists because it follows a bottom-up testing method. This method tests the leaves of the GO graph, and then it removes all genes annotated at significant child-terms from the parent-term’s gene list before testing the terms whose descendant have already been tested.

### Evaluation of the methodology (Figure 1B)

We validated our methodology using two gold standards; each one was based on an independently published list of biological processes (GO terms) enriched in differentially expressed genes derived from microarray experiments of cancer stroma microdissected out of whole tumors [2,3]. The Fisher’s exact test was performed between the GO terms of (i) each “gold standard” and those (ii) overrepresented among the “Xhyb genes”. This was based on the assumption that between the xenograft model and the human environment, the stromal response to human cancer has a shared phenotype leading to a shared Gene Ontology annotation. Thus, the stromal response in xenograft models could be applied to cancer study. In particular, the first gold standard **(GS1)** contained 61 biological processes in the differentially expressed breast stromal genes that were identified by comparing laser microdissected stromal tissues (adjusted to cancer) with paired normal epithelium [3]. The second gold standard **(GS2)** compared gene expression profiles of breast tumor stroma captured by laser capture microdissection, and reported the overrepresented GO terms associated with poor-prognosis, good-prognosis and mixture-outcome, respectively [2]. These 107 biological processes derived from the whole tumor with poor prognosis were used as the second gold standard because samples with poor prognosis are more representative for characterization of stromal signatures [2]

The network of the enriched GO biological process terms and those in the two gold standards were visualized using Bioconductor packages *Rgraphviz* and *GOstats.* An additional evaluation for the union of statistically significant GO terms(*p*-*value*<1%, unadjusted cumulative conditional hypergeometric test) were performed as described in (Additional file 1: Suppl. Method and Additional file 2: Suppl. Table 2. The Xhyb probes and R script can be downloaded from http://www.lussierlab.org/publications/Stroma.

## Results

### Identification of Xhyb probes

After a sequence similarity search of a human probe with mouse RNA sequence databases and the subsequent biological cross-species expression experiment, cross-species hybridizing probes were identified and additional species-specific probes were designed by our research group for the same gene. As shown in the left plot of **Figure D** in Additional file 1: Suppl. Methods, we examined the top 18% (notated as BGS2.18) of the absolute expression of mouse probes when exposed to human RNA because it had the highest F-score (precision=40%, recall=25%) together with the 3^rd^ theoretical prediction model (noted as M-Xhyb3). We examined multiple conditions in the M-Xhyb3 and developed an optimized model (right plot of Figure D, Additional file 1: Suppl. Methods). Using this optimized model, we adjusted the parameter, *x*, of BGS2. This process resulted in 6,200 Xhyb probes involving 5,300 mouse genes from GPL7202 and 5,900 Xhyb probes involving 4,900 human genes from GPL6480, respectively.

### Xhyb genes found in published deregulated genes from xenograft models

The identified Xhyb subset of the literature-reported tumor deregulated genes is listed in Additional file 2: S. Table 1. Note that there were many genes targeted by multiple Agilent probes. In such cases, reported genes with only one cross-mouse hybridizing probe were annotated as “Xhyb deregulated genes” while reported genes with both Xhyb and none-Xhyb probes were considered as “possible Xhyb deregulated genes”. Details are given in Additional file 2: S. Table 3-4. Furthermore, among the 17 identified cross-mouse hybridizing human probes that were reported by literature in Additional file 2: S. Table 3, 13 probes targeted their mouse homologs (Additional file 2: S. Table 5) as predicted by the BLASTN algorithm [17] with default parameters. Several biological processes associated with stromal cells were significantly (p<1%) enriched only among the Xhyb deregulated genes. For example, it has been previously shown that implanted tumors developed intensive angiogenesis with vascular endothelial growth factor (*VEGF*) induction in the stroma [21]. Accordingly, we found that positive regulation of vascular endothelial growth factor receptor signalling pathway was significantly enriched among the Xhyb deregulated genes (*p*=0.004), suggesting a different stromal/tumor cell cross-talk and was further demonstrated by differences in angiogenesis and in necrosis [19].

### Evaluation

**Figure 3:**
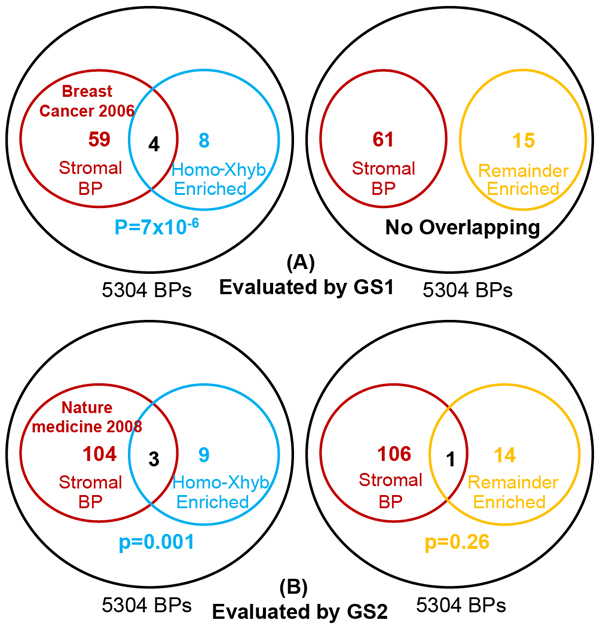
**Comparison of the gold standards **[2,3]** of stromal biological process (BP)**. The BP terms enriched among the genes with cross-hybridizing probes that target mouse homologs (Homo-Xhyb), and among the remaining genes for glioblastoma deregulation [19] were respectively compared with gold standards. **Legend**: a, b: two sample groups; c: effect of interested; i: probe id; FC: fold change (**Equation 1**).

The biological process terms associated with cross-hybridizing probes that targeted mouse homologs (**Methods, Enrichment d**) significantly overlapped with the known stromal gold standard terms as defined in **Methods, Evaluation of the methodology** section. As shown in Figure 3, four out of twelve GO terms overlapped with the GS1 (Fisher’s Exact Test *p*=7x10^-6^). In contrast, none of the 15 GO terms enriched among remaining non-cross-hybridized genes (**Methods, Enrichment c**) matched the GS1 (Figure 3A). Examining the second gold standard, three Xhyb genes enriched GO terms (**Methods, Enrichment d**) overlap with GS2 (Fisher’s exact tested *p*=0.001). On the other hand, there was only one overlap from the remaining gene enriched GO terms (**Methods, Enrichment c,***p*=0.26) (Figure 3B). Moreover, an alternative evaluation focusing on the genes with cross-hybridizing probes (**Methods, Enrichment b**) also resulted in significant overlap between the two gold standards (*p-value*=0.07 and 0.01 respectively) for Xhyb gene enriched GO terms. Noteworthy, there was no overlap between Xhyb gene enriched GO terms (**Methods, Enrichment b** and **Enrichment d**) and the whole tumor annotations from the subset with good prognosis. This finding corroborates the hypothesis that biological process annotations associated with poor prognosis contain more stromal components.

**Figure 4:**
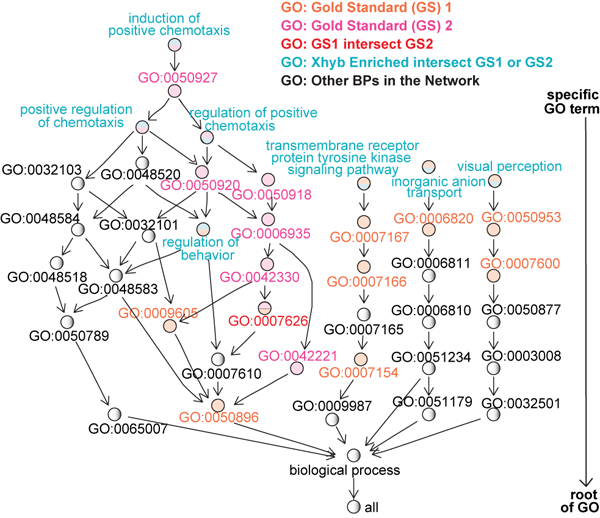
**The network of the Xhyb enriched BP terms and two gold standards.** A full network including all identified BP terms are given in Additional file 3: Suppl. Figure 1

Figure 4 visualizes the graphical relationship of the identified BP terms and two gold standards (Additional file 2: S. Table 6). The biological process, “regulation of behaviour”, is of interest as a root term in 4 out of the 7 identified terms that also have overlap with GS2. This may suggest that tumor stromal actions or reactions are in response to external or internal stimuli through the regulation of chemotaxis. Note that 11 out of the 12 identified GO terms are the child nodes of the stromal gold standard terms (Additional file 3: Suppl. Figure 1).

In addition, as presented in Additional file 4: S. Figure 2 panel a, deregulated Xhyb genes **(Enrichment d,** cyan circles) are significantly better at predicting biological processes of stromal cells when compared to the the remaining genes (**Enrichment c**) and the full reported gene list (**Enrichment a**) annotations in both studies. Conversely, Additional file 4: S. Figure 2 panel b shows that the remaining deregulated genes (**Enrichment c,** orange triangles) or the full gene list (**Enrichment a,** blue squares) are better at predicting biological processes associated to cancer cells.

## Discussion

**Limitations and future studies.** This study focused on human probes that were inadvertently cross-hybridized with mouse RNAs. The confounded expression of 5,000 human and mouse genes can thus be assessed; however, these measures are limited in that they are not genome-wide. The platform-specific cross-species hybridizing probes, thus, limits our study as we are indirectly measuring the stromal microenvironment effects as compared to the direct measurements that could arguably be obtained with species-specific mouse probes.

**For future studies,** we plan to use knowledge extracted from the literature to further refine the interpretation using BioMedLEE [22] in a high-throughput manner. Our current experiment identified Xhyb probes in Agilent human microarrays and therefore can only reveal part of the stromal signatures. A more complete view of stromal signatures in xenograft model should be investigated using mouse-specific probes. To further identify microenvironment factors associated with tumor progression and clinical prognosis, we are currently developing a novel methodology to dissect cancer and stromal signature using a xenograft model of head and neck cancer.

## Conclusions

In this paper, we introduced a novel design to detect biological processes of the stroma using human arrays with probes of which approximately 25% cross-hybridized with mouse genes. We also conclude that the majority of our identified cross-hybridized genes target their corresponding homolog. Although human and mouse RNA gene expression are confounded in this subset of probes, we have demonstrated that an appropriately performed Gene Ontology enrichment analysis can identify significant stroma-associated GO terms by evaluating our findings with GO annotations from previous laser capture microdissections of the stroma. These results suggest that our method may be a reasonable alternative to laser microdissection in a specially designed chip. Our results also suggest that human chip probes cross-hybridizing with mouse genes are, in fact, over-annotated with stroma-associated biological processes. This leads us to believe that xenograft gene expression contain confounded cell signals and stromal cell signals which must be accounted for. An additional benefit of this methodology is that from a cost perspective, this *in silico* approach allows us to extract additional stromal knowledge from current xenograft tumor arrays without needing to perform further *in vivo/in vitro* experiments. In future studies, we intend to examine these cross-species enrichments for biological interactions between cancer cells and stromal cells, an important initiative of the National Cancer Institute Tumor Microenvironment Think Tank [23].

## Competing interests

The authors declare that they have no competing financial or other interest in relation to the work reported here.

## Authors' contributions

YAL supervised experiment design, computational methods, results and the manuscript redaction; XY conducted the majority of the analyses of data; YL performed the Blasten programming; HY conducts the online custom Agilent array design; HRX designed and supervised the biological experiments; YAL, HRX and YH contributed to the biological interpretation of the results; everyone contributed to the interpretation of the results. All authors read and approved the final manuscript.

## Supplementary Material

Additional file 1 Click here for file

Additional file 2 **S. Table 1:** Xhyb subset of previous xenograft studies reported cancer deregulated genes.
**S. Table 2:** The significant (p<1%) GO terms among the deregulated genes in a published glioblastoma xenograft model.
**S. Table 3:** Xhyb subset of the differential expressed genes in a previous xenograft study and their corresponding probes in the Agilent array (GPL6480).
**S. Table 4:** Xhyb subset of the 28 deregulated genes reported by a previous colon tumor xenograft study and their corresponding Agilent array (GPL6480) probes. Genes in bold are those identified as Xhyb. According to the author (Gouye *et al* 2008), HT-29-derived 5M21 which displayed a constitutive invasive behavior in type I collagen, while TATI was shown in this study to be a major autocrine and transforming factor produced by HT-29-derived 5M21 cells to control colon cancer cell invasion and metastasis.
**S. Table 5:** The human probes in S. Table 3 that target their mouse homologs.
**S. Table 6:** The identified stromal associated GO terms (hypergeometric test *p-value*<1%) that also reported by two gold standards using laser microdissection technology. Click here for file

Additional file 3 Significantly enriched (*p*<1%) BP terms among the genes with mouse homologous cross-hybridizing probes, and their overlapping with two gold standards. The identified BP terms with their nearest parent-terms of the gold standard are listed below the network graph. Click here for file

Additional file 4 Comparison of the overrepresented biological processes (BPs) derived from three GO en-richment tests for the reported gene list. Different data points are the proxy recall and precision under a serial of thresholds for GO enrichment test. ** Panel a** shows that in predicting stromal processes, the reported Xhyb subset of deregulated genes performs better than either the remain-ing genes or the full reported gene list, since only the precision-recall predictions from reported Xhyb genes (cyan circles) are significant (proxy *p*<5%). **Panel b** shows that the Xhyb subset of deregulated genes are the worst at predicting cancer processes compared to the other two kinds of gene lists with lower precision and recall. Click here for file
