# Supplementary material for: Stromal microenvironment processes unveiled by biological component analysis of gene expression in xenograft tumor models
Source: BMC Bioinformatics. 2010 Oct 28;11(Suppl 9):S11. doi: 10.1186/1471-2105-11-S9-S11 (PMC2967741; doi:10.1186/1471-2105-11-S9-S11)
Supplement: Additional file 1 [file 1471-2105-11-S9-S11-S1.pdf]

Stromal Microenvironment Processes Unveiled by Biological Component Analysis of Gene Expression in Xenograft Tumor Models
(Supplementary Methods)

Xinan Yang, PhD[1]*, Younghee Lee, PhD[1]*, Yong Huang, MD, MSc[1], James L Chen, MD[2], H. Rosie Xing, PhD[3,4 §] and Yves A Lussier, MD[1,4,5 §]

Data: Multiple data resources are used in this study as listed in the following **Table A**.

Table A. Data resources used in this study.

Content	Data source	Data version (downloaded data)
The GO annotation for both human and mouse genes	NCBI (ftp://ftp.ncbi.nlm.nih.gov/gene/DATA/gene2go.gz)	Sep. 22, 2009
The mouse homolog of human genes	NCBI (ftp://ftp.ncbi.nih.gov/pub/HomoloGene)	Build63
Probe annotation for Agilent 44k whole human genome array	GPL6480 from GEO (http://0-www.ncbi.nlm.nih.gov.millennium.unicatt.it/projects/geo/query/acc.cgi?acc=GPL6480)	on Sep., 2009
Possible mouse target of probes in human array	Agilent company	May, 2008
78 genes differentially expressed between the first and later GBM xenograft generations.	PNAS (2006) 103(44) 16466-16471	
28 genes deregulated in K18Y_TATI mutant tumor xengrafts versus the control 5M21 xenografts.	Oncogene (2008) 27: 4024-4033	
The mapping between gene symbol, GenBank ID and GO annotation for human gene	"org.Hs.eg.db" from Bioconductor	Version 2.2.0

Array Preprocessing. One slide of the Agilent whole genome 4x44k microarray with two dual-arrays was used. The total pooled RNA was amplified and labeled according to the Agilent Low RNA input Fluorescent Linear Amplification Kit protocol. Human RNA and mouse RNA were

labeled with Cy5/red in two arrays, respectively; and the pooled human and mouse RNAs were labeled with Cy3/green. A mount of 1.5 ug labeled RNA per channel (1.5 ug human RNA or 1.5 ug mouse RNA in Cy5, while 0.75 ug human RNA pooled with 0.75 ug mouse RNA in Cy3) was hybridized onto GPL6480 microarray for 17 hours, according to the Agilent's two color quick amp labeling protocol. The two arrays were scanned with 5 um resolution and 100% power gain at 600PMT for both Cy3 & Cy5 using the GenePix 4000B axon scanner. Subsequent data was extracted by the Agilent Feature Extraction Software.

Assumption Verification. We performed gene ontology enrichment studies that were conducted over cross-species hybridizing probes of the human array to produce a set of GO terms pertaining to the biological processes of the stroma. There are two assumptions that need to be verified:

Assumption A) Majority of the Xhyb Probes Designed for Human Genes Target their Homologous Mouse Genes. To verify this assumption, we estimated the proportion of homologs between human and mouse in two cases: **a)** all the 19,725 genes in the whole Agilent human microarray and **b)** the identified 4,948 human genes with cross-hybridizing probes.

To this end, we attained two data resources. **1)** Parts of possible mouse targets for all human probes in the Agilent 44k whole human genome microarray were provided by Agilent Company (http://www.lussierlab.org/publications/Stroma/SupplTable9.xls), containing 5,791 unique mouse genes with GenBank accession number. **2)** The human and mouse homologous database downloaded from NCBI contains 16,715 unique human genes with mouse homologous genes. Among them, there are 94% (15,689) of human homologous genes having probes in the Agilent 44k whole human genome microarray.

First, the majority of the possible cross-species hybridizing happens between homologous genes, because that 74% (4,266) of the possible mouse targets are the homologous ones of their designed human targets. **Second**, the majority of our identified Xhyb genes target their homolog. This is because that 40% of the possible homologous targets (n=1,701) are identified as Xhyb, while only 26% of the possible homologous targets (n=3,247) are among the remainders in our experimental result. As shown in **Figure A,** this result suggests a significant enrichment between the possible homologous cross-hybridization and the identified genes with cross-hybridizing probes (Fisher's exact tested p-value<$2x10^{-16}$).

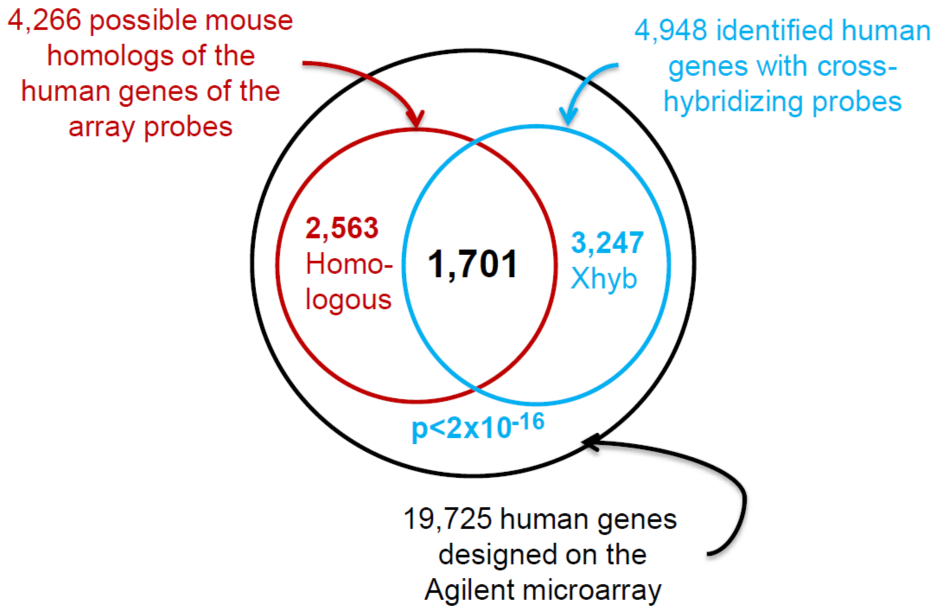

Figure A. Majority of the Xhyb probes designed for human genes target the homologous mouse genes.

Assumption B) Majority of the Gene Ontology Annotations for human and mouse homologous genes are identical. As presented in **Figure B** and **Table B** concerning GO annotation, for 17,355 pairs of homologous genes between human and mouse that were downloaded from NCBI (version 63), 83% pairs (14,339) have identical GO annotations. On the view of genes, there are 17,920 mouse genes with GO annotations of which 83% (14,803) are

human homologs. And 80% (14,298) of mouse genes have a GO Annotation that is identical to the GO Annotation of its human homologos. Meanwhile, there are 17,688 human genes with GO annotations and 86% (15,223) of these human genes have mouse homologos. The GO annotations of 80% (14,167) of all the human genes are identical with the GO annotations of their mouse homologous.

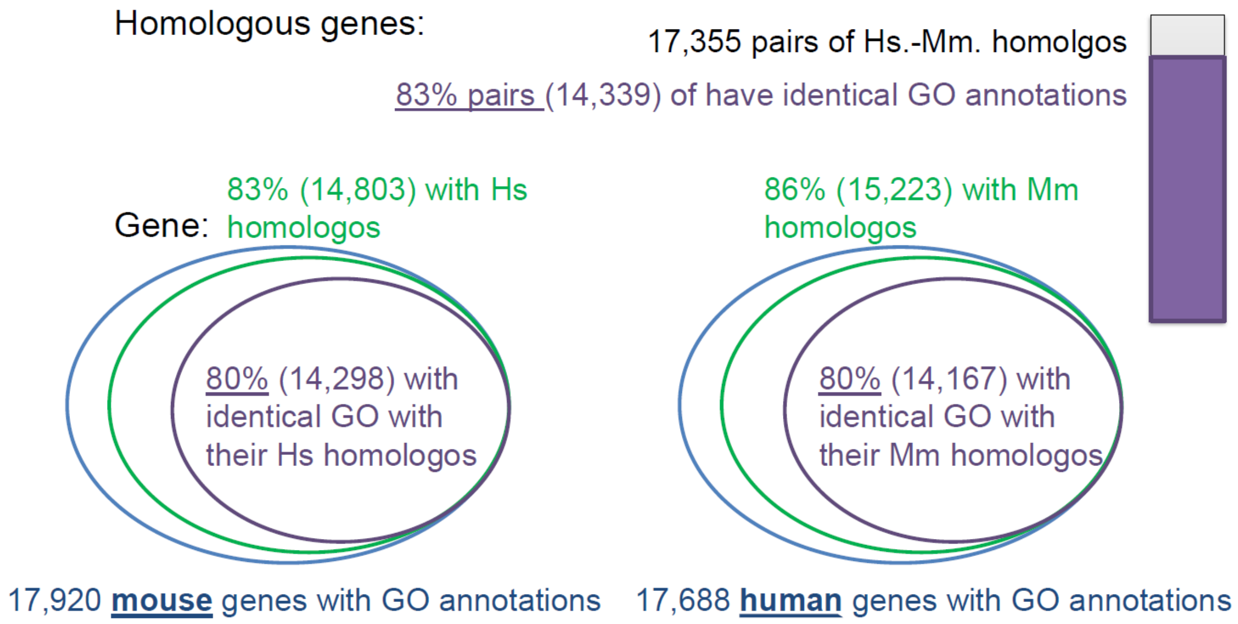

Figure B. The proportion of genes with identical GO annotations to those of their homologs.

Table B. The counting of GO-gene occurrences and distinct genes considering homologs.

	**A: NCBI GO annotation**		**(A) intersect (NCBI homologs)**		**Identical**		**Not Identical**	
	GO-gene occurrence	Distinct genes	GO-gene recurrence	Distinct genes	GO-gene occurrence	Distinct genes	GO-gene occurrence	Distinct genes
Hs	141,809	17,688	128,930	15,223	82,448	14,167	46,482	2,728
Mm	142,139	17,920	124,555	14,803	82,448	14,298	42,107	2,931

Sequence similarity searching for cross-hybrid chip design. The standalone Basic Local Alignment Search Tool (BLAST) software version 2.2.14(J Mol Biol 215: 403-10 (1990)) was downloaded from ftp://ftp.ncbi.nlm.nih.gov/blast/ and was installed on a Solaris machine.

FASTA format of the human and mouse RefSeq sequences were separately formatted by the formatdb program for the blast database. The Blastn program was executed to search for sequence similarity of a probe as a query against target RNA sequence database with default blast options (mouse cross-hybrid probes against human genes or human cross-hybrid probes against mouse genes). BL2SSEQ was run to compare the two sequences between a probe and a target.

Array Analyses and cross-species hybridizing (Xhyb) Probe Identification (Figure C). The background subtracted signal derived by Agilent Feature Extraction was log2-transformed. After excluding the technical control probes, 34k human probes and 33k mouse probes expressed in the green channel were selected for further study using a criteria that required the expression intensity to be larger than zero on the log scale value. The identification of Xhyb probes for the human array and the mouse array was conducted utilizing the following six steps, where we presented the way to identify mouse probes that are most likely to hybridize with human RNA as example: **Step 1:** To predict cross-species hybridized probes, the Blast program was performed to explore a sequence similarity between human probe, mouse mRNA and vice-versa with default Blast options. **Step 2:** Biological gold standards (**BGS**) are given in **Table C** in order to capture the mouse probes whose targets express highly when exposed to human RNA. **Step 3:** Theoretical prediction models were given in **Figure D** and **Table D** considering different sequence alignment conditions. **Step 4:** In order to optimize the parameters in the above theoretical models, precision, recall and their F-scores were calculated for four biological GSs against four theoretical models (**Figure D left**) where $F = 2 \cdot \frac{precision \cdot recall}{precision + recall}$. After selecting the best BGS that balances the precision and recall, the possible combinations of Blast predicting conditions were further optimized according to F-measurement (**Figure D right**). **Step 5:** The

parameter of the best biological GS was further decided according to the combination of the optimal blast conditions. **Step 6:** Finally, the Xhyb probes were identified using the optimal BGS.

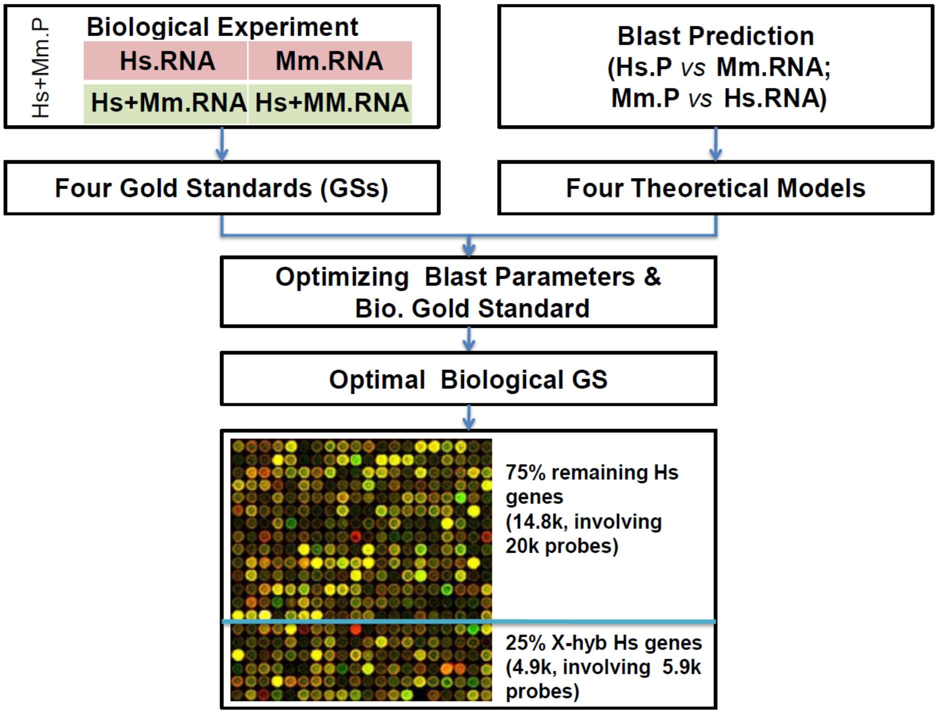

Figure C. Array analyses and cross-species hybridizing (Xhyb) probes identification.

As a result, the optimal blast parameters are: {18<Align≤20 and mis<6} Union {20<Align≤30 and mis<7} Union {30<Align≤40 and mis<8} Union {40<Align≤45 and mis<9} Union {45<Align≤50 and mis<10} Union {50<Align and mis<11}.

The corresponding F-score for each biological gold standard (**BGS**) against each model is given in the left plot where different lines represent different BGSs and the different numbers represent the different theoretical models. Per the plot, 1) biological GS 2.18 together with M-Xhyb3 achieves the highest F-score and 2) M-Xhyb 3 always has a higher F-score compared with other models. In the right plot, multiple Blast conditions were separately learned to get the optimal theoretical model.

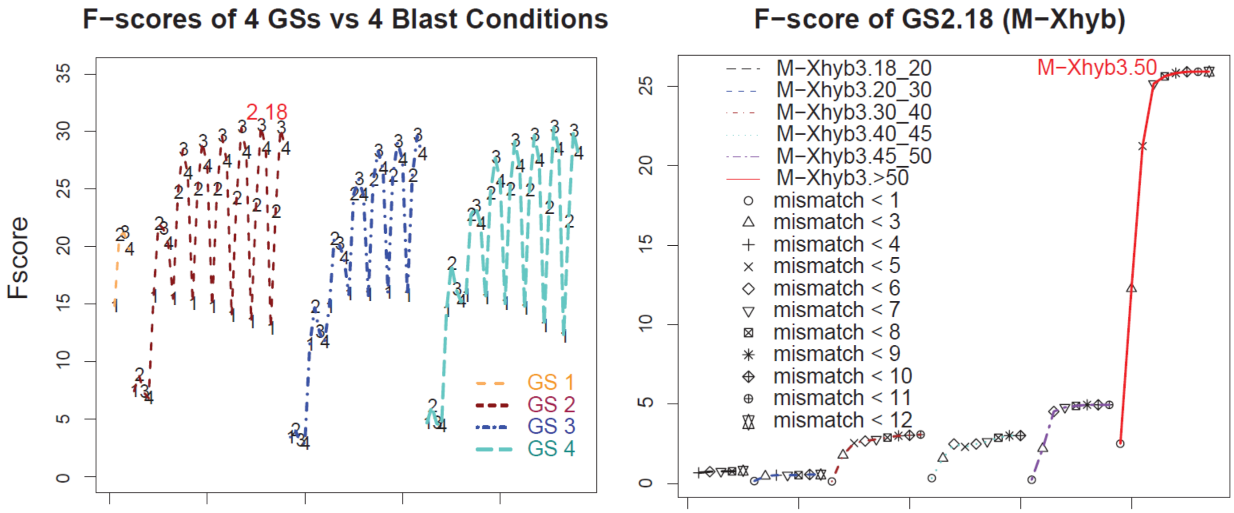

Figure D. Optimizing Blast parameters and biological gold standards.

Table C. Definition of four biological GSs to predict Xhyb probes.

BGS	Parameters
BGS1.*x*	top *x* ratio of relative expression (red/green) when exposed to wrong RNA vs to correct RNA:
BGS2.*x*	top *x*% absolute expression when exposed to human RNA with mouse probe
BGS3.*x*	top *x*% relative expression when exposed to human RNA with mouse probe
BGS4.*x*	the same as GS2 but absolute log2 based expression on both green channels of Hs+Mm RNA expression of mouse probes > 4

Table D. The details of the four models for theoretical prediction.

Models	Conditions and parameters
M-Xhyb1	score > 70 & Alig > 58 & mis < 6
M-Xhyb 2	Alig > 50 & mismatches < 6
M-Xhyb 3	{18<Align≤20 and mis<5} U {2<Align≤ 30 and mis<6} U {30<Align≤40 and mis<7} U {40<Align≤50 and mis<8} U {50<Align and mis<9}
M-Xhyb 4	{15 < Align ≤ 30 and mis < 2} U {30 < Align ≤ 40 and mis < 3} U {40 < Align ≤ 50 and mis < 5} U {50< Align and mis < 7}

Legend: Align: alignment; mis: mismatch; U:union.

Additional Evaluation. Next, the union of GO terms that were statistically significant (unadjusted conditional hypergeometric tested *p-value*<0.01) by any one of our three enrichment tests were classified into four groups by a cancer expert. These four GO term groups were:

stromal/normal function-related, cancer cell related, neither stromal nor cancer cell related and unknown (**S. Tab. 2**). Based on this limited data, we calculated the precision for stromal cell related terms (*i.e.* the proportion of stromal cell terms that were associated to the deregulated Xhyb genes) and proxy recall (*i.e.* the proportion of deregulated Xhyb genes enriched GO terms among the classified stromal terms). Only proxy recall could be estimated because no gold standard for all GO terms concerning stromal cells were available at the time of prediction. To increase robustness, serial thresholds of significance were used (0.9%, 0.8%, 0.7%, 0.6%, 0.5%) for the above precision-proxy recall calculation. Moreover, the possibility to predict the stromal cell terms among the classified GO terms by chance was estimated for each above threshold using the Bioconductor package *verification*.
