# Supplementary material for: Stromal microenvironment processes unveiled by biological component analysis of gene expression in xenograft tumor models
Source: BMC Bioinformatics. 2010 Oct 28;11(Suppl 9):S11. doi: 10.1186/1471-2105-11-S9-S11 (PMC2967741; doi:10.1186/1471-2105-11-S9-S11)
Supplement: Additional file 2 — S. Table 1: Xhyb subset of previous xenograft studies reported cancer deregulated genes. S. Table 2: The significant (p<1%) GO terms among the deregulated genes in a published glioblastoma xenograft model. S. Table 3: Xhyb subset of the differential expressed genes in a previous xenograft study and their corresponding probes in the Agilent array (GPL6480). S. Table 4: Xhyb subset of the 28 deregulated genes reported by a previous colon tumor xenograft study and their corresponding Agilent array (GPL6480) probes. Genes in bold are those identified as Xhyb. According to the author (Gouye et al 2008), HT-29-derived 5M21 which displayed a constitutive invasive behavior in type I collagen, while TATI was shown in this study to be a major autocrine and transforming factor produced by HT-29-derived 5M21 cells to control colon cancer cell invasion and metastasis. S. Table 5: The human probes in S. Table 3 that target their mouse homologs. S. Table 6: The identified stromal associated GO terms (hypergeometric test p-value<1%) that also reported by two gold standards using laser microdissection technology. [file 1471-2105-11-S9-S11-S2.pdf]

Stromal Microenvironment Processes Unveiled by Biological Component Analysis of Gene Expression in Xenograft Tumor Models
(Supplementary Tables)

Xinan Yang, PhD[1*], Younghee Lee, PhD[1*], Yong Huang, MD, MSc[1], James L Chen, MD[2], H. Rosie Xing, PhD[3,4§] and Yves A Lussier, MD[1,4,5§]

Supp. Table 1: Xhyb subset of previous xenograft studies reported cancer deregulated genes.

Cancer Type	Xhyb deregulated Genes	Possible Xhyb deregulated genes
Glioblastom[illegible] (GBM)[19]	*CLIC1, COL11A1, CRYAB, EMP3, GPR17, HTRA1, ID1, ITPKB, MAG, PTTG1, RAB33A, SERPINE2, SGK1, VEGFA*	*PDGFRA, INSM1, IGFBP3, CCND1, TMPO, C5orf13, PSME1, CDKN1A, TJP2,PPP3R1 , DDR1, B2M, SCARA3, EMP1, GFAP*
Colon cancer[6]	*IGFBP2, IGF2, FN1, TAGLN, ZBTB16, PRKCA, ID8-72*	*HPN, TFF3*

Supp. Table 2: The significant (p<1%) GO terms among the deregulated genes in a published glioblastoma xenograft model.

GOTERM	Classification			Conditional hypergeometric p-value		
	N	S	C	Full List	Xhyb genes	Remainder genes
enzyme linked receptor protein signaling pathway				0.0084	0.0031	
positive regulation of vascular endothelial growth factor receptor signaling pathway		1			0.0038	
negative regulation of cellular process				0.0066	0.0043	
negative regulation of transforming growth factor beta receptor signaling pathway				0.0004	0.0076	
cartilage condensation		1			0.0076	
BMP signaling pathway		1	1		0.0076	
negative regulation of BMP signaling pathway		1	1		0.0076	
positive regulation of chemotaxis		1	1		0.0076	
regulation of positive chemotaxis		1	1		0.0076	
induction of positive chemotaxis		1	1		0.0076	
visual perception	1				0.0088	
phosphoinositide-mediated signaling				0.0015		0.0003
nervous system development			1	0.0025		0.0011
anatomical structure development						0.0019
mesenchymal cell development		1		0.0063		0.0023
signal transduction		1	1	0.0000		0.0023
cell-cell signaling		1	1			0.0042
dTMP biosynthetic process				0.0052		0.0046
deoxyribonucleoside monophosphate biosynthetic process		1	1	0.0052		0.0046
pyrimidine deoxyribonucleoside monophosphate metabolic process		1	1	0.0052		0.0046
positive regulation of phosphoinositide 3-kinase cascade				0.0052		0.0046
positive regulation of cyclin-dependent protein kinase activity				0.0001		0.0091
posterior midgut development	1					0.0091
cyclin catabolic process			1			0.0091
pyrimidine deoxyribonucleotide biosynthetic process						0.0091
clathrin cage assembly						0.0091
transmembrane receptor protein tyrosine kinase signaling pathway		1	1	0.0003		
cell growth		1	1	0.0005		
multicellular organismal development				0.0006		

regulation of phosphate metabolic process				0.0009
transforming growth factor beta receptor signaling pathway		1	1	0.0024
regulation of protein amino acid phosphorylation				0.0026
cell differentiation		1	1	0.0039
negative regulation of phosphorylation				0.0039
organ development	1			0.0042
response to stress		1	1	0.0043
positive regulation of non-apoptotic programmed cell death			1	0.0052
regulation of protein kinase activity				0.0060
phosphorus metabolic process				0.0064
regulation of transferase activity				0.0071
regulation of cell growth		1	1	0.0072
extracellular structure organization and biogenesis		1	1	0.0081
regulation of biological quality		1	1	0.0082

Legend: N: neither cancer nor stromal cell process; S: stromal cell or functional process; C: cancer cell process; blank in classification: unknown; blank in conditional hypergeometric p-value: not significant (p>1%).

Supp. Table 3: Xhyb subset of the differential expressed genes in a previous xenograft study and their corresponding probes in the Agilent array (GPL6480).

Genes in bold are those identified as Xhyb. According to the literature (Sakariassen et al. 2006), the tumors became more vascular and circumscribed with emerging necrotic regions in the sub-sequence generation. Moreover, MRI scans showed less invasive, strongly contrast-enhancing tumors in the higher generation.

		Corresponding Agilent Probes				Biological
**Symbol**	**FC**	**ID**	**Annotation**	**GENE**	**SYMBOL**	**V1Anno**
B2M	-4	A_23_P37441	NM_004048	567	B2M	Xhyb
		A_24_P740662	AK022379	567	B2M	
C5orf13	2.4	A_24_P149124	NM_004772	9315	C5orf13	Xhyb
		A_32_P98423	NM_004772	9315	C5orf13	
CCND1	3.2	A_24_P193011	NM_053056	595	CCND1	Xhyb
		A_23_P202837	NM_053056	595	CCND1	
		A_24_P124550	NM_053056	595	CCND1	
CDKN1A	-2.8	A_23_P59210	NM_000389	1026	CDKN1A	Xhyb
		A_24_P89457	NM_078467	1026	CDKN1A	
**CLIC1**	-2.9	A_23_P30884	NM_001288	1192	CLIC1	Xhyb
**COL11A1**	2.5	A_23_P11806	NM_080629	1301	COL11A1	Xhyb
**CRYAB**	-6.2	A_24_P206776	NM_001885	1410	CRYAB	Xhyb
DDR1	-3.9	A_24_P123601	NM_013993	780	DDR1	Xhyb
		A_23_P93311	NM_013993	780	DDR1	
		A_24_P367289	NM_013993	780	DDR1	
EMP1	-7.6	A_23_P76488	NM_001423	2012	EMP1	Xhyb
		A_24_P921446	BC017854	2012	EMP1	
**EMP3**	-2.4	A_23_P119362	NM_001425	2014	EMP3	Xhyb
GFAP	-7.8	A_24_P31165	NM_002055	2670	GFAP	Xhyb
		A_23_P66593	NM_002055	2670	GFAP	
		A_24_P59786	NM_001131019	2670	GFAP	
**GPR17**	-2.8	A_23_P16963	NM_005291	2840	GPR17	Xhyb
**HTRA1**	-2.5	A_23_P97990	NM_002775	5654	HTRA1	Xhyb
**ID1**	-10.5	A_23_P252306	NM_002165	3397	ID1	Xhyb
IGFBP3	3.4	A_23_P215634	NM_001013398	3486	IGFBP3	Xhyb
		A_24_P320699	NM_001013398	3486	IGFBP3	
INSM1	3.4	A_24_P31676	NM_002196	3642	INSM1	Xhyb
		A_23_P109184	NM_002196	3642	INSM1	
**ITPKB**	-3	A_23_P372255	NM_002221	3707	ITPKB	Xhyb
**MAG**	-4.2	A_24_P279704	NM_080600	4099	MAG	Xhyb
PDGFRA	13.1	A_23_P300033	NM_006206	5156	PDGFRA	Xhyb
		A_23_P332536	BC015186	5156	PDGFRA	
		A_32_P100379	AA599881	5156	PDGFRA	
PPP3R1	-3.3	A_23_P108592	NM_000945	5534	PPP3R1	
		A_24_P388252	NM_000945	5534	PPP3R1	Xhyb
PSME1	-2.3	A_23_P151610	NM_006263	5720	PSME1	Xhyb
		A_23_P151614	NM_006263	5720	PSME1	

**PTTG1**	2.1	A_23_P7636	NM_004219	9232	PTTG1	Xhyb
**RAB33A**	-3.1	A_23_P147025	NM_004794	9363	RAB33A	Xhyb
SCARA3	-4.9	A_23_P215900	NM_016240	51435	SCARA3	Xhyb
		A_24_P232158	NM_182826	51435	SCARA3	
**SERPINE2**	2.6	A_23_P50919	NM_006216	5270	SERPINE2	Xhyb
**SGK1**	-2.5	A_23_P19673	NM_005627	6446	SGK1	Xhyb
TJP2	-3.1	A_23_P9293	NM_004817	9414	TJP2	Xhyb
		A_24_P201153	NM_201629	9414	TJP2	
TMPO	2.5	A_24_P210244	NM_001032283	7112	TMPO	Xhyb
		A_23_P325040	NM_003276	7112	TMPO	
**VEGFA**	8.9	A_23_P70398	NM_001025370	7422	VEGFA	Xhyb
		A_23_P81805	NM_001025366	7422	VEGFA	Xhyb
		A_24_P12401	NM_001025366	7422	VEGFA	Xhyb
		A_24_P179400	NM_001025370	7422	VEGFA	

Supp. Table 4: Xhyb subset of the 28 deregulated genes reported by a previous colon tumor xenograft study and their corresponding Agilent array (GPL6480) probes.

Genes in bold are those identified as Xhyb. According to the author (Gouye *et al* 2008), HT-29-derived 5M21 which displayed a constitutive invasive behavior in type I collagen, while TATI was shown in this study to be a major autocrine and transforming factor produced by HT-29-derived 5M21 cells to control colon cancer cell invasion and metastasis.

			Corresponding Agilent Probes				
**GenBank no.**	**Symbol**	**Ratio**	**ID**	**GB_ACC**	**GENE**	**SYMBOL**	**Bio. Anno.**
NM_177978	CHRD	-5.2	A_23_P502047	NM_003741	8646	CHRD	
			A_24_P117725	NM_003741	8646	CHRD	Xhyb
NM_00212482	**FN1**	-2.8	A_24_P119745	NM_212482	2335	FN1	Xhyb
			A_24_P85539	NM_212482	2335	FN1	Xhyb
NM_00182983	HPN	-2.1	A_23_P101806	NM_182983	3249	HPN	Xhyb
			A_23_P406782	NM_182983	3249	HPN	
NM_002166	**ID2**	2.4	A_23_P143143	NM_002166	3398	ID2	Xhyb
			A_32_P69368	NM_002166	3398	ID2	Xhyb
NM_000612	**IGF2**	-4.6	A_23_P150609	NM_000612	3481	IGF2	Xhyb
			A_23_P421379	NM_000612	3481	IGF2	
NM_000597	**IGFBP2**	-9.6	A_23_P119943	NM_000597	3485	IGFBP2	Xhyb
NM_002737	**PRKCA**	2.2	A_23_P55099	NM_002737	5578	PRKCA	Xhyb
			A_24_P916496	NM_002737	5578	PRKCA	Xhyb
NM_001001522	**TAGLN**	-2.2	A_23_P87011	NM_001001522	6876	TAGLN	Xhyb
			A_23_P87013	NM_001001522	6876	TAGLN	Xhyb
NM_003226	TFF3	2	A_23_P257296	NM_003226	7033	TFF3	Xhyb
			A_23_P393099	NM_003226	7033	TFF3	
			A_24_P289208	NM_003226	7033	TFF3	
NM_006006	**ZBTB16**	2	A_23_P104804	NM_006006	7704	ZBTB16	Xhyb

Supp. Table 5: The human probes in S. Table 3 that target their mouse homologs.

ProbeID	Hs. gene	Hs. symbol	Anno	Mm. Homolog	Mm. gene	Target*	Score *	Homo-Xhyb**
A_23_P30884	1192	CLIC1	Xhyb	Clic1	114584	AK150382	88.33	Y
A_23_P11806	1301	COL11A1	Xhyb	Col11a1	12814	AK157734	93.62	Y
A_24_P206776	1410	CRYAB	Xhyb	Cryab	12955	NW_001030907.1	91.5	Y
A_23_P119362	2014	EMP3	Xhyb	Emp3	13732	NW_001030849.1	78.8	Y
A_23_P16963	2840	GPR17	Xhyb	Gpr176	381413			N
A_23_P97990	5654	HTRA1	Xhyb	Htra1	56213			N
A_23_P252306	3397	ID1	Xhyb	Id1	15901	NT_039207.7	62.8	Y
A_23_P372255	3707	ITPKB	Xhyb	Itpkb	320404			N
A_24_P279704	4099	MAG	Xhyb	Mag	17136	NT_039413.7	62.6	Y
A_23_P7636	9232	PTTG1	Xhyb	Pttg1	30939	XR_002209	92.73	Y
A_23_P147025	9363	RAB33A	Xhyb	Rab33a	19337	NM_011228.1	86	Y
A_23_P50919	5270	SERPINE2	Xhyb	Serpine2	20720			N
A_23_P19673	6446	SGK1	Xhyb	Sgk1	20393	BC070401	96.67	Y
A_23_P70398	7422	VEGFA	Xhyb	Vegfa	22339	NM_001025250	96.67	Y
A_23_P81805	7422	VEGFA	Xhyb	Vegfa	22339	NM_001025250	90.48	Y
A_24_P12401	7422	VEGFA	Xhyb	Vegfa	22339	NM_001025250	98.33	Y
A_24_P179400	7422	VEGFA		Vegfa	22339	NM_001025250	104	Y

Legend: Anno: Annotation; Xhyb: cross-mouse hybridize; Homo: homologous; HomoXhyb: the human probes target its mouse homogous predicted by BLASTN.

* The reported target and score using BLASTN Algorithm with Default Parameters (http://www.ncbi.nlm.nih.gov/genome/seq/BlastGen/BlastGen.cgi?taxid=10090)

** Y refers to BLASTN reports significant alignment, otherwise N.

Supp. Table 6: The identified stromal associated GO terms (hypergeometric test *p-value* <1%) that also reported by two gold standards using laser microdissection technology.

GOID	Biological Process	Test Method	Reported Gold Standard
GO:0007167	enzyme linked receptor protein signaling pathway	test b	GS1- Breast Cancer Res. 2006
GO:0007601	visual perception	test b, test d	GS1- Breast Cancer Res. 2006
GO:0015698	inorganic anion transport	test d	GS1- Breast Cancer Res. 2006
GO:0050795	regulation of behavior	test d	GS1- Breast Cancer Res. 2006
GO:0007169	transmembrane receptor protein tyrosine kinase signaling pathway	test d	GS1- Breast Cancer Res. 2006
GO:0050926	regulation of positive chemotaxis	test d, test b	GS2 - Nat. Med 2008
GO:0050930	induction of positive chemotaxis	test d, test b	GS2 - Nat. Med 2008
GO:0050921	positive regulation of chemotaxis	test d, test b	GS2 - Nat. Med 2008

Test d and test b are conditional hypergeometric tests, using the same background gene list, all the identified genes with cross-hybridize probes on the array. But test b uses the deregulated genes with identified hybridizing probes as inputted gene list, while test d filters the list into homologous genes with Xhyb probes (see **Methods**).
