# Supplementary material for: Stromal microenvironment processes unveiled by biological component analysis of gene expression in xenograft tumor models
Source: BMC Bioinformatics. 2010 Oct 28;11(Suppl 9):S11. doi: 10.1186/1471-2105-11-S9-S11 (PMC2967741; doi:10.1186/1471-2105-11-S9-S11)
Supplement: Additional file 3 — Significantly enriched (p<1%) BP terms among the genes with mouse homologous cross-hybridizing probes, and their overlapping with two gold standards. The identified BP terms with their nearest parent-terms of the gold standard are listed below the network graph. [file 1471-2105-11-S9-S11-S3.pdf]

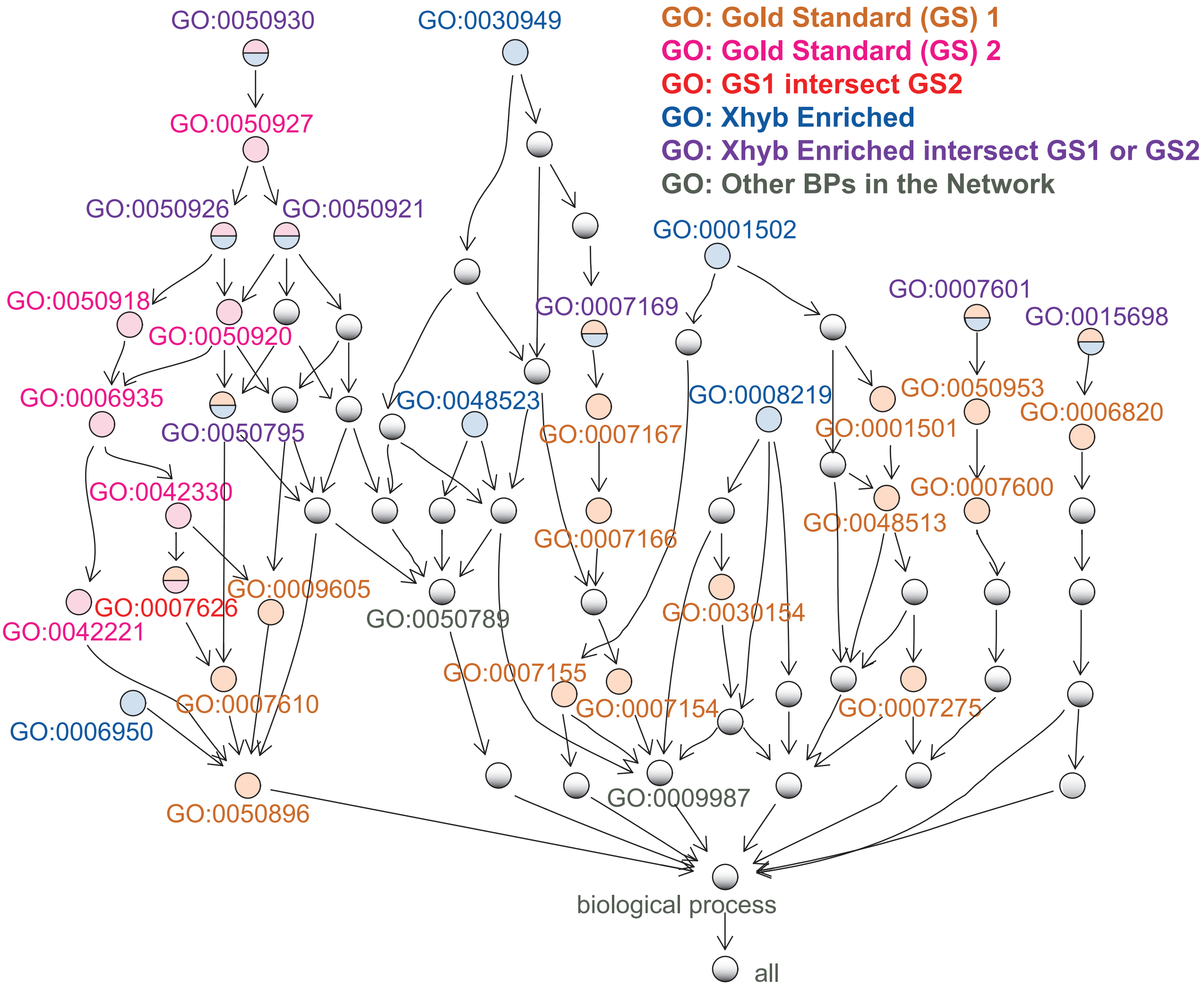

ID	Identified Term	Nearest GS ID	Nearest GS Term	Shortest Distance*
GO:0030949	positive regulation of vascular endothelial growth factor receptor signaling pathway	GO:0007169	transmembrane receptor protein tyrosine kinase signaling pathway	3
GO:0008219	cell death	GO:0030154	cell differentiation	2
GO:0001502	cartilage condensation	GO:0007155	cell adhesion	2
GO:0006950	response to stress	GO:0050896	response to stimulus	1
GO:0048523	negative regulation of cellular process			

*: Number of between Nodes +1
