# Supplementary figures and images for: Stromal microenvironment processes unveiled by biological component analysis of gene expression in xenograft tumor models
Source: BMC Bioinformatics. 2010 Oct 28;11(Suppl 9):S11. doi: 10.1186/1471-2105-11-S9-S11 (PMC2967741; doi:10.1186/1471-2105-11-S9-S11)

**a) Stromal GO**

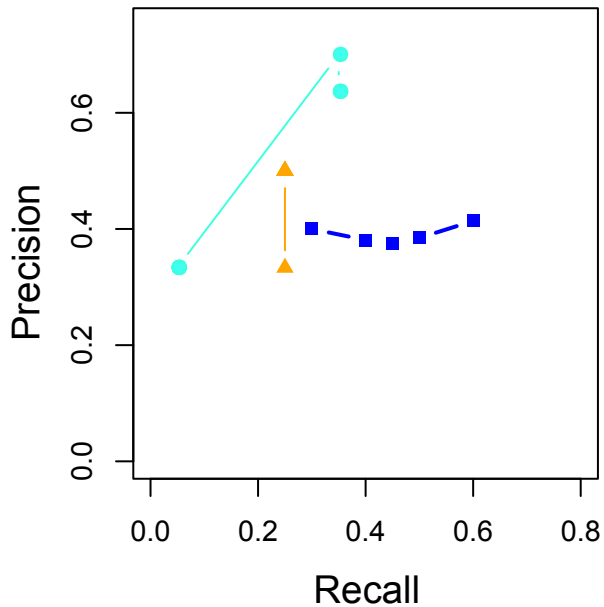

**b) Cancer GO**

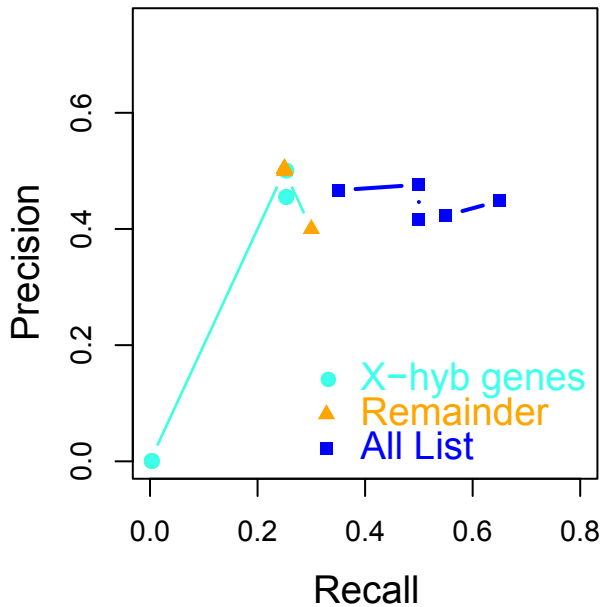

Supplement: Additional file 4 — Comparison of the overrepresented biological processes (BPs) derived from three GO en-richment tests for the reported gene list. Different data points are the proxy recall and precision under a serial of thresholds for GO enrichment test. Panel a shows that in predicting stromal processes, the reported Xhyb subset of deregulated genes performs better than either the remain-ing genes or the full reported gene list, since only the precision-recall predictions from reported Xhyb genes (cyan circles) are significant (proxy p<5%). Panel b shows that the Xhyb subset of deregulated genes are the worst at predicting cancer processes compared to the other two kinds of gene lists with lower precision and recall. [file 1471-2105-11-S9-S11-S4.pdf]
